# How Built Environment Characteristics Influence Social Interactions During Neighbourhood Walks Among Urban Inhabitants

**DOI:** 10.3390/ijerph21111519

**Published:** 2024-11-15

**Authors:** Sina Kuzuoglu, Troy D. Glover, Luke Moyer, Joe Todd

**Affiliations:** Department of Recreation and Leisure Studies, University of Waterloo, 200 University Avenue Waterloo, Waterloo, ON N2L 3G1, Canada; troy.glover@uwaterloo.ca (T.D.G.);

**Keywords:** placemaking, right to the city, urban walking, built environment, health and wellbeing

## Abstract

As an accessible and low-risk mode of transportation and recreational activity, walking both produces and is produced by socio-spatial urban features. The health benefits of walking transcend physical fitness, remaining integral to mental health and to fostering social connectedness in urban communities. Understanding what drives walking behaviour, therefore, warrants attention from a public health perspective. This qualitative case study focuses on the social interactions of inhabitants during neighbourhood walks and how built environment features influence walking patterns and experience. Using diaries, maps, and semi-structured interviews with 45 inhabitants of a mid-sized Canadian city, this research investigates the influence of permanent and temporary physical features on the perceived quality of inhabitants’ walks. The findings show the public visibility of urban modifications influences walking behaviour and improves social interactions, leading to a heightened sense of belonging and community. Inhabitant-led modifications in the urban space were mostly neighbourhood-bound and voyeuristic, whereas administrative interventions were more successful for collectivization. Both types of interventions are argued to foster social connectedness through different mechanisms, with positive impacts on inhabitants’ health and wellbeing. The findings underscore the relevance of community-led and administratively planned interventions in built environments in positioning public health policies associated with social cohesion and connectedness.

## 1. Introduction

Upon leaving the privacy of our urban dwellings, we greet the familiarity of our neighbourhoods. This familiarity, while evident in the built structures we encounter, including the designs of streets and the architectural styles of the houses surrounding us, also extends to the people we meet, namely our neighbours and familiar passers-by. However, as cities grow and people adopt a more individualistic lifestyle, particularly in the Global North, the concept of neighbouring and one’s engagement with one’s neighbourhood as a physical space shift significantly. The severe public health restrictions on individual mobilities and constraints on social interactions during the COVID-19 pandemic, however, reminded us of the importance of the relationships we develop with people in close proximity to ourselves [1].

Neighbourhood walking is not only a physical activity, but also a social one—two aspects that complement each other in acting to improve health both on an individual and on a collective level [2,3,4,5]. Therefore, the surge in neighbourhood walking as an important everyday activity we recently witnessed [6] has significant potential for addressing public health challenges of growing social isolation and loneliness pervading across the world [7]. This paper positions neighbourhood walking as a complex relationship between the built environment and the social interactions urban form supports within neighbourhoods, a relationship that was seemingly amplified (or simplified) during the pandemic.

In this context, the neighbourhood represents a physical and a social phenomenon. This interplay between the physical and the social produces the neighbourhood as a social space [8]. When engaged in neighbourhood walking, urban inhabitants not only move through an abstract physical space, but also occupy their neighbourhood’s social infrastructure, including its streets, parks, and squares [9,10,11]. These spaces simultaneously serve to connect private and public realms. Accordingly, when we physically navigate a neighbourhood’s streets, we engage within a semi-private setting, not necessarily because we are situated on or next to a private property, but rather because we are visible to the private owners of the space, and vice versa. Therefore, in a strictly technical sense, one can rarely be fully immersed “in public” in the neighbourhood unless one does not belong to that neighbourhood. Alternatively, one can describe unfamiliarity with neighbours as the co-presence of people when “physically proximate, but socially distant” ([12], p. 608).

To be sure, urban walking is a sociable practice (e.g., [6,13,14]); however, how small and/or temporary modifications to the built environment are perceived and experienced arguably shapes the sociability in and of urban walking [15,16], and therefore culminates in “enduring effects” ([17], p. 618), an area that remains relatively underdeveloped in scholarship. The purpose of the study, therefore, was to explore how various physical and social conditions within neighbourhoods impact the walking experiences and community connections of inhabitants. This study aimed to understand the significance of both the built and natural environments, along with community-driven initiatives, in shaping perceptions of walkability and fostering social interactions among neighbours. By examining these dynamics, this research seeks to contribute to a deeper understanding of how walkability not only influences individual health and wellbeing, but also enhances community cohesion and resilience in urban settings and contributes to a public health agenda. To these ends, we employed a mix of purposive and snowball sampling to reach the “socializers” of neighbourhoods, i.e., those individuals who maintained a comparatively large social network in their neighbourhoods and were willing to discuss neighbourhood-related matters [18], as well as to connect us with other socializers. This sampling approach partially relied on urban inhabitants’ social networks—combined with neighbourhood-level demographic characteristics—to support the collection of in-depth information about inhabitants’ neighbourhood walks.

Our data collection included walking diary entries, walking maps, and semi-structured interviews with residents of Kitchener-Waterloo (KW) in Ontario, Canada, focusing on participants’ observations of general physical conditions [19] and changes in the built environment [16] during their neighbourhood walks. We aimed to understand how the features of the urban environment influenced their social interactions and relationships, as mediated by their walks. The changes encountered and the positive interactions they facilitated emerged as a significant influence on urban inhabitants’ walking patterns, thereby underscoring the significance of neighbourhood walking as a physical and social activity that fosters belonging, social connectedness, and ultimately health and wellbeing.

## 2. Literature Review

The review of the literature that follows begins with a focus on the production of urban space, exploring urban planning as a political process, whereby those in power design spaces to align with their visions, while also recognizing the success of these spaces depends on how inhabitants interact with, perceive, and appropriate them. Next, we examine how walking plays a key role in shaping the relationships between urban inhabitants and their environment, serving as both a physical and a social activity that encourages interaction, supports public health, and is influenced by the built environment’s diversity. The final section reviews the social dimension of the relationship between walkability and built environments. Together, these sections provide the foundation for understanding social interactions within the built environments of urban neighbourhoods and their implications for health and wellbeing.

### 2.1. Producing Urban Space

Urban planning is an inherently political process that embodies the politically favoured vision of the material city and social organization within. Lefebvre [8] viewed this process as the social production of space in which those with power over the material space (i.e., the conceived space) aim to generate a politically favourable version of what the space seemingly affords (i.e., the perceived space). The conceived and perceived spaces, however, evolve in a dialectical tension between how inhabitants interact within a space and how they experience it (i.e., lived space). A Lefebvrean spatial dialectic outlines the mechanisms with which the conceived space aims to homogenize the social attributes of the space and subsumes the resisting forces of the lived space [20]. Thus, the space is not static, but rather continuously evolving. How and the extent to which space is modified—either in its conception, or perception, or how it is lived—shape the (re)production of space. As Koch and Latham [15] reminded us, modifying the urban space requires us to think not only within the boundaries of the material space and its design, but also to consider how such modifications resonate in the relationships among urban inhabitants and the built and natural environments.

In this view, imposing certain physical characteristics onto a space does not necessarily lead to the intended social outcomes. This observation also echoes in discussions on the aesthetic dimension of urban planning in which the subjectivity involved in assessing aesthetics makes it difficult to reconcile the representative vision and the actual life this vision would facilitate [21]. Therefore, one may argue spatial planning frameworks that are flexible over how inhabitants may appropriate urban space (see also [22]) according to their desires and expectations [23] would potentially alleviate parts of this disconnection. Lefebvre [24] viewed urban inhabitant-led processes of appropriation as integral to the right to the city, alongside the inhabitants’ right to participate in decision-making processes concerning the production of urban space (see also [25]). Urban inhabitants’ direct involvement in changing their cities potentially contributes to playfulness of and in cities, which manifests in the relationship between inhabitants and the built environment [26].

In this view, both administratively imposed and inhabitant-led changes to the urban space—denoted by such concepts as tactical urbanism [27] and the animation of spaces [16,17]—have an imminent reflection on the urban fabric and the relationalities among those occupying the same urban space. As Smith [17] argued, the primary aim of such modifications is to influence the interactions between individuals and introduce new forms of relationalities into the urban space. These new relationalities emerge through both the changes in how a space is perceived by its regular users and the new users such modifications invite. In other words, every change in urban spaces creates a unique public with unique modes of interactions and relationalities. The decisive factor in the “success” of urban modifications is not solely where the changes are implemented, but rather the extent to which urban inhabitants acknowledge them and consider them aligned with their expectations [26]. These modifications are, in essence, stimulants for urban inhabitants that enhance their attentiveness to their surroundings in their pedestrian mobilities [28]. Therefore, one may argue navigating cities by walking provides urban inhabitants with opportunities to explore their neighbourhoods (and cities) and the modifications enacted in urban spaces, thereby potentially influencing how they interact with others [29].

### 2.2. Urban Walking and (Public) Health

How people move within and explore the urban is paramount to understanding the relationships cultivated with urban environments and the social beings who inhabit them. De Certeau [30] viewed walking as “an elementary form of [the] experience of the city” (p. 93). The early conceptualizations of urban strollers, or flâneurs, focused on how the modernization process affected human behaviour in cities [31], where urban strollers were primarily seen as wandering the urban streets with “fleeting and dispersed attention” [13] (p. 307) as they engaged with the diversity of the city [32]. In this sense, one ought to question whether strolling in limited diversity, such as that which occurs in familiar surroundings (i.e., the neighbourhood) can actually be considered flânerie.

Contemporary investigations of urban walking differentiate primarily between purposeful walking (e.g., getting somewhere or walking as a sport/ exercise) and leisure (recreational or pleasure) walking (e.g., [3,19]). As Giles-Corti et al. [4] asserted, each urban walk can be characterized as a unique combination of its utilitarian, leisure, and social dimensions, underlining the multifaceted nature of urban walking. This multifaceted nature undergirds our understanding of urban walks as physical and social activities when suggesting that—in all its combinations—through walking, inhabitants are “discovering, creating and or transforming the city” ([33], p. 136) (see also [34]).

Walking remains a low-risk and accessible physical activity for a relatively high proportion of inhabitants for contemporary cities [2]. In a recent review article, Kelly et al. [35] synthesized the benefits of walking on physical (e.g., body composition, cardiovascular disease, and blood pressure) and mental health (e.g., positive mood and cognitive functioning) and underscored the importance of future studies to scale up walking-related research to be operationalized on a public health level. As Nordh et al. [36] suggested, “walking … can be adapted to different needs and situations … with a great potential to increase public health” (p. 64), and they pointed toward the role of built environment and how it was conceived in influencing walking behaviour and supporting active lifestyles. However, the physical activity of walking is rarely just a physical activity. Accordingly, Green [37] urged scholars to more cautiously evaluate the public health potential politically ascribed to pedestrian mobilities, as these physical activities are entangled with social conditions and (in)equalities, with a clear influence on who is (or, more crucially, not) welcome in urban spaces.

The socially constructed limitations imposed on urban walking, particularly toward contextually marginalized groups such as visible and/or ethnic minorities or tourists, that might physically or symbolically exclude them from walking and/or be present in urban public spaces [37] suggest that the practice of urban walking might differ between social groups. For example, if individuals do not feel an association or a belonging to their neighbourhood’s broader social expectations, they may restrict themselves from being co-present with others in the neighbourhood’s public realm. However, such exclusionary practices do not diminish the importance of the associated microsociology to the everyday practice of urban walking. Some examples include not only the tension between pedestrians in how they navigate the urban space [14] or the rude behaviours encountered [38], but also more convivial interactions with fellow urbanites that range from fleeting encounters—i.e., sociable co-presence that provide a sense of security through the familiarity of strangers (e.g., [39])—to interactions marked by civil attention during casual interactions [6]. Such fleeting and/or casual interactions have the potential to develop into stronger, more meaningful relationships through persistent exposure in familiar contexts [9,40], notwithstanding potential interactional barriers [29]. As this multitude of relationalities that emerge when engaged in neighbourhood walking implies, the mechanisms that may support the development of social connections during walks vary significantly.

Urban settings are physically and socially diverse, and this diversity in the built environment and among urban inhabitants allows for different relationships to emerge, which may support social connectedness in different ways. As Leavell et al. [41] observed, for example, in urban areas, social structures can be strengthened through contact with natural resources, with an expected improvement in overall physical and mental health in communities. The importance of public spaces, such as parks, became particularly visible to urban inhabitants during pandemic-related restrictions [42,43]. While previous research underlined the wide-ranging positive effect of access to urban green spaces on mental health on both the individual and the population-based levels [44,45,46], it is important to note the mostly quantitative nature of this body of literature. Hence, the subjective understandings of the built environment and green spaces for urban residents offer a potential complementary avenue to explore in our aim to connect built environment features, neighbourhood walking, and physical and mental health.

Regarding the social connectedness aspect, which is particularly relevant for social cohesion, there are nuanced findings among different demographic groups. For example, while Franke et al. [47] found increased physical activity, including walking and cycling, through community programs is associated with lower levels of loneliness among older adults, recent research on adolescents’ physical activity in public recreational spaces did not indicate a correlation between physical activity and social connectedness [48]. The different spatial and social parameters in these studies point toward the complex interplay of the built environment and social determinants in supporting social connectedness in communities. Therefore, the role of the built environment in influencing walkability, and thereby urban inhabitants’ interactions, is imperative to address.

### 2.3. Walkability and the Built Environment

Research related to walkability tends to centre on formal planning processes and the decisions made by local authorities. This emphasis stems from the practical understanding that policy and planning directly shape walkable environments, which subsequently influences public health outcomes. A significant body of literature identifies the key attributes that contribute to walkability, including street connectivity [49,50], land use mix [51], residential density [51], the quality of sidewalks [52], and the availability of local amenities [53]. These factors are often quantitatively assessed in studies that examine how the built environment can foster increased physical activity levels among urban inhabitants.

While these studies contribute valuable insights on the built environment-related factors, they tend to overlook the broader context of the social, environmental, and individual factors that significantly influence walkability. For example, inhabitants’ preferences for walkable neighbourhoods reflect not only physical attributes, but also social dynamics that foster community engagement and social interactions [53,54]. The physical features of spaces are reflected in the social interactions they facilitate, which ultimately contribute to the meaning-making processes of urban inhabitants concerning these spaces and, more broadly, their neighbourhoods. Furthermore, a community’s characteristics and their connection to the neighbourhood are important contributors to its perceived safety and desirability [55], rendering these features’ implementation solely through administrative measures difficult to realize. Walkable neighbourhoods often encourage greater community involvement and a sense of belonging among residents, which enhances overall wellbeing [53].

Despite the recognition of these complex interdependencies, the dominant narrative in walkability research remains primarily focused on local authorities and their roles in shaping urban environments. This gap is critical to address, as it limits the understanding of how diverse influences, including personal experiences and community-driven changes, contribute to the perception and reality of walkability. Scholars are increasingly advocating for a more holistic approach that integrates both top-down planning perspectives and bottom-up community initiatives [26].

Our study aligns with this emerging discourse by emphasizing the necessity of considering a range of factors beyond formal planning decisions. Through qualitative insights gathered from participants, we aimed to reveal how social interactions, familiarity with the environment, and community engagement shape the walking experience, offering a more nuanced perspective on the complexities of walkability. In doing so, we hope to contribute to the ongoing conversation around effective public health strategies that promote active living in urban spaces. This nuanced understanding of walkability served as the foundation for our methodological approach, which prioritized participants’ experiences and perspectives in examining the interplay between the built environment and social dynamics.

## 3. Material and Methods

This paper is part of a larger research project that aimed to gain a deeper understanding of how urban inhabitants’ neighbourhood walks are associated with their social interactions and their sense of social connectedness. It focuses on the built environment, namely how physical features influence neighbours’ walking patterns and sense of social connectedness as they relate to individual and public health of communities. While there is considerable scholarly interest in the association between the built environment and walking, it commonly involves employing a quantitative approach (e.g., [56,57]). A similar observation also applies to research on social ties where the objective properties of social networks are utilized to formalize their structures [58,59]. This formalization using objective data often results in an abstraction of social life [60], and therefore relegates differential perceptions of urban inhabitants with regard to their social interactions to secondary importance. In our attempt to gain a deeper understanding of the social dynamics involved, this paper uses a qualitative approach in its exploration of the social experiences of urban inhabitants when they walk in their neighbourhoods [61] and their association with the built environment. While qualitative data analysis inevitably involves researchers’ interpretation—introducing potential subjectivity and bias—it also employs rigorous analytical techniques and methodological approaches designed to enhance the trustworthiness of findings, which we achieved through peer debriefing [62].

The geographical setting of this research was the mid-sized twin cities of KW in Ontario, Canada, with a community of over 400,000 inhabitants located about one hundred kilometres southwest of Toronto. KW offers a mix of suburban and high-density neighbourhoods that combine older, established, and newer developments, thereby providing a dynamic environment in which to explore how both physical infrastructure and social conditions shape walking experiences and community connections. Due to this geographical focus, purposive sampling—based on “[participants’] relevance to the research question, analytical framework, and explanation or account being developed in the research” ([63], p. 232)—started through advertisement of the project via a local newspaper and radio, which included a brief outline and a link to a web page with additional information and a sign-up page. In line with the aim of obtaining detailed accounts of social life facilitated through neighbourhood walking, it was imperative to reach urban inhabitants who Felder [18] described as “socializers”, i.e., those who not only maintain social relations with a comparatively large number of their neighbours, but also are enthusiastic and active in connecting with their neighbours and willing to discuss it. Partially because of these attributes, the socializers of a neighbourhood were expected to both provide detailed information about their neighbourhoods and connect us with other inhabitants whose insights could further contribute to our understanding of neighbourhood dynamics. In this sense, purposive sampling was combined with snowball sampling to recruit participants (see [64]). These conventional strategies of recruitment for qualitative research resulted in 45 inhabitants of KW participating in our study between September 2021 and June 2022. However, sampling employed in this research led to some homogeneity among our participants—particularly in terms of gender, ethnicity/ race, age, and education—which can be observed in Table 1.

Upon the completion of the demographic information survey, data collection took place in three successive stages. First, the participants were asked to keep diaries of at least five of their neighbourhood walks in which they were expected to provide information on the general characteristics of their walks (i.e., weather, length, and duration) and to reflect on their walks (i.e., motivation/ purpose; walking environment; people they saw/encountered; people with whom they interacted and how; and whether they walked alone, with someone else, or a pet). While diaries are commonly employed in walking studies—particularly to collect statistical information [65]—qualitative diaries are especially valuable as they encourage participants’ to be more conscious of their walks and provide an insight into their experiences (e.g., [14]). In total, our participants completed 240 diary entries. A sample diary entry is provided in Table 2.

In the second stage, we asked the study participants to (1) indicate perceived neighbourhood boundaries, (2) draw their walking routes (in different colours if more than one), and (3) identify the locations where they saw meaningful people (i.e., friends, acquaintances, and familiar strangers) using an online map platform. We expected these qualitative maps to help us contextualize the participants’ walks and to help them focus and reflect [66] and to contribute to the internal consistency of the data and improve the trustworthiness of the interpretation by allowing the triangulation of the spatial data with qualitative information [67]. While the qualitative spatial analysis of the information provided in the maps was originally planned, we were only able to elicit maps from 21 participants. Therefore, for the respondents who provided the maps, the information was incorporated into the third stage of data collection as a supplement to the walking diary entries.

The final part of data collection consisted of semi-structured interviews with our participants. This stage allowed our participants to provide the context and additional insights on their experiences in neighbourhood walking [68]. Both the diaries and the maps were reviewed prior to the interviews—which were coordinated and conducted online by research assistants—and the information provided in them were utilized to help the participants recall the experiences they described in the preceding stages. Interviews were recorded and transcribed. The interviews started with the participants’ motivation for walking (and whether the pandemic had any impact on their motivations) and their social interactions during their walks with the intention of understanding different relationalities, such as verbal and non-verbal interactions (e.g., conversations or nodding relationships), who they recognized or routinely encountered without knowing (e.g., friends, acquaintances, or familiar strangers) [18]. In this stage, we also inquired about walking’s perceived role in getting to know more about the socio-spatial characteristics of neighbourhoods. The information provided in the maps (if applicable) proved to be useful in gaining additional insight into the social parameters of neighbourhood walks [69]. Second, our participants were asked to describe the processes that resulted in the emergence of the social ties they identified in the preceding stages (e.g., reciprocity) and the role neighbourhood walking played in this process. This stage also incorporated differential aspects of the built environment as noted by participants (e.g., “very closed off front,” or “front porch [people]” [from Alistair’s interview]) as they relate to building social relationships. The final part of the interviews focused on the material and non-material resources (i.e., neighbourhood support mechanisms) to which these social ties provided access and concluded with participants’ reflection on their neighbourhood walks after partaking in our research. The list of interview questions is provided in Table 3.

The ethics approval for this research was obtained from the University of Waterloo. The collected data were stored in an online repository accessible to the members of the research team, which facilitated the researchers’ familiarity with and immersion in the entire data set [70]. Once each research team member was familiar with the collected information, data analysis was structured as a collaborative iterative process of content analysis with a two-stage coding process that resulted in the identification of common themes present in the data relevant to the built environment focus of this paper (as the second-level theme). The first-level themes identified in the analysis stage are (1) walkability and physical conditions; (2) the features of the built environment (e.g., public spaces, architecture, and the urban planning features of neighbourhoods); (3) changes in the built environment and animation; (4) the types of social interaction during neighbourhood walks (e.g., nodding, greeting, and interactions with familiar strangers, acquaintances, friends, and family). To ensure the anonymity of the research participants, we used pseudonyms in our reporting. The Findings Section that follows focuses on the role the built environment plays in how our participants experience their neighbourhoods and (in some cases) the urban area of KW during their walks.

## 4. Findings

The findings reported in this section are organized thematically. To begin, the participants’ perceptions regarding the physical conditions of their walks are reported, followed by the temporary changes they observed in their walking routes, and ending with the participants’ observation of their social interactions in their neighbourhood space. The data reported from the interviews are noted with an I, while the data from the diaries are noted with a D.

### 4.1. Physical Conditions of the Walks

Because the participants were recruited for this research throughout the calendar year, the data reported in the walking diaries provided insights into the seasonality of their walks. Not surprisingly, the participants who completed diaries in winter months were particularly observant of the physical conditions of their walking routes. Notwithstanding the common concern of ill-maintained sidewalks, some people noticed routine neighbourhood walkers—in that they became familiar strangers—which led to their increased attention to the physical conditions of pathways. The concern for neighbourhood walkers’ health and wellbeing and the associated camaraderie resulted in friendly exchanges between neighbours, such as “I wanted to make sure I got the sidewalk shovelled before you came” (Patricia, I), or “we cleared the sidewalk for you. What are you doing on the other side of the street?” (Elizabeth, I).

The recipients of such thoughtful gestures associated this behaviour with feelings of community in the neighbourhood. Olivia (I) described an instance when she shovelled the driveway of “the guy who does everybody’s sidewalk [on their street] … after he [got sick]”. In this context, attentiveness to the physical conditions of the walking routes was regarded as a positive neighbourly attribute that contributed both to the quality of the walking experience and to community-building efforts. For spaces under the purview of municipal services, perceived maintenance for improved walkability varied. For example, the “urban trails are well maintained and excellent … [but the] city sidewalks are terrible and icy” (Lilly, D). Such observations amplified the perceived safety and appeal of walking for many of the participants recruited during winter months.

### 4.2. Temporary Changes on Walking Route

Aside from the general physical conditions observed by our participants, three main subthemes emerged with respect to temporary changes in the neighbourhood and on the walking routes of our participants, which can be broadly associated with (1) natural elements, (2) bottom-up urban interventions, and (3) top-down changes and structured programming.

First, the presence of natural elements—particularly those deemed visually striking—was an important factor that improved neighbourhood walking. Thus, the participants viewed the landscaping in urban public spaces as important. Well-groomed natural(-ized) spaces were noticed and appreciated by the participants in times of seasonal change. As Grace (D) recognized “some summer flowers have been replaced by fall mums. So well cared for by City employees.” The autumn time, with the changing colours of tree leaves and seasonal flowers, represented a particular highlight for the inhabitants of the KW area. The diary entries were replete with descriptions of this natural (and cyclical) beautification process. Most of our participants noticed the changes in the natural environment, which, at times, influenced their walking route: “the fall colours are breath-taking, so I figured I would go walk on a trail” (Audrey, D). This behaviour was also relevant to major urban green spaces (e.g., legacy parks) with “Victoria Park [becoming] very picturesque” (Juliet, D). In addition to public green spaces, the neighbours’ efforts to beautify the publicly visible parts of their own houses were also noted by our participants as something that led to an increased awareness of and appreciation for “the beautification that nature brings to us” (Dorian, I).

Second, our participants pointed to various bottom-up efforts to improve their neighbourhoods. One of our participants highlighted small changes in the neighbourhood in what they called “art in the wild”, such as “painted stones around the base of a tree … or a chalkboard on the street … that bolster[ed] morale … [made] the walk a little more interesting” (Ruth, I). Such changes were also used to commemorate more solemn events, such as the “purple ribbons … in memory of the [deceased] 15-year-old girl” (Elizabeth, D). Such instances were examples to bottom-up urbanism; however, they mostly positioned the participants in a passive role as the observers of bottom-up interventions. Some changes, however, invited inhabitants to actively participate in the modified built environment, like “the printed activities for kids [on the sidewalk] … with direction to tell people to jump, run, walk, etc., on them” (Iris, D).

Related to these bottom-up efforts were the decorations that adorned many houses during culturally significant dates, such as Christmas and Halloween. Such occasions had the potential to transcend their voyeuristic tendencies (i.e., people decorating their houses) to become communal efforts, such as the neighbourhood organization of “the light festival [to build] the Santa’s runway” that showed the “playfulness about where we live” (Sylvia, I). Other efforts, such as neighbourhood parties, supported similar experiences of community attachment. Moreover, the availability of events in public spaces was considered important; those “in the parks or by the school … definitely build that sense of community … where you can go to meet people … and [have] shared experiences so that you’re not strangers” (Sylvia, I).

Third, in addition to inhabitant-led small-scale changes, neighbourhood walks allowed the inhabitants to encounter public art and other programmed activities in various locations in the city and attracted them to participate. For example, the participants recalled “hear[ing] music from a house and unexpectedly [coming] across a small concert,” (Florence, D), “a new art installation [at the Library]” (Margaret, D), and the “[mobile] Oktoberfest Gemutlichkeit wagon … folks who had gathered responded to the music, doing the chicken dance” (Penelope, D). Our participants mostly viewed such activities in a positive light and were particularly attentive to publicly visible arts like the “funky and quirky metal art … in front of several homes … always brings a smile” (Grace, D). Expectedly, not all artistic endeavours were well received; Liam (D), for example, questioned the presence of the “super ugly stack of orange sticks making an art installation”.

In sum, the unexpected or previously unknown changes that broke the routine walking experience, unsurprisingly, changed the way the participants perceived their neighbourhood and walking routes. Although not all such experiences took place in a social setting, they nevertheless had an indispensable role in facilitating neighbourly interactions, potentially leading to stronger community connections.

### 4.3. Interacting in the Neighbourhood

Neighbourhood walking was viewed as a way to keep track of the changes that occurred in the neighbourhood and led to the development of a sense of one’s neighbours through these changes. Most of our study participants agreed that walking allowed them to remain social. Since COVID-19, regular walkers noted encountering a higher number of walkers on their routes. COVID-19 also had an impact on how people moved when walking insofar as “everyone’s doing the swerve for you [and] walk on the road and you’re not getting close to other people” (Alistair, I). Whereas some viewed this new walking etiquette in a positive light (i.e., “respectfully stay[ing] away” [Florence, D]), to some, it was “avoidance behaviour that makes no scientific sense [and were] frustrated with the matter” (Miranda, I).

How the neighbourhood was physically structured had an important role in influencing the relationalities and interactions in the neighbourhoods of KW. While some of our participants alluded to the possibility to obtain a sense of one’s neighbours through such things as “the signs on their lawns … [or] the big flag on the back of [their] vehicle” (Reginald, I), even in the absence of any direct interactions, physical features were highlighted. Regarding the neighbourhoods’ built environment characteristics, our participants commonly viewed houses with front porches as facilitators of more frequent (albeit casual and, to some extent, superficial) interactions between neighbours and walkers. For Margaret (I), “the downtown and the old neighbourhoods, they’re front porch neighbourhoods. People don’t hide out in their backyard … [which is] much more interactive.” A similar observation was also made by a younger participant: when “it’s not porch seasoning [sic]… [one] can’t strike up conversations much” (Violet, I).

In this context, the relatively limited visibility of backyards was seen as a factor in restricting relationships from developing. However, the physical structure of the backyards also had the potential to improve interactions among neighbours. Maintaining the visibility of one’s backyard was recognized by one participant as an important factor in developing a closer relationship with their next-door neighbours through “talking over [their low] fence” (Ursula, I). As Alistair (I) noted, in some neighbourhoods, the neighbours did not maintain a physical separation of their backyards where “the kids would … run through all the backyards and all the parents … knew each other. It was really neat”.

## 5. Discussion

This study explored the intricate relationship between neighbourhood characteristics and positive social interactions during neighbourhood walks, highlighting the implications for individual and public health. Our findings indicate that the built environment features significantly influence social interactions during routine walks, thereby emphasizing the importance of familiar surroundings in promoting physical activity. Notwithstanding the possibilities of negative interactions in neighbourhoods (e.g., [38]), the participants mostly reported feeling a heightened sense of safety through their positive interactions with their neighbours and passersby while walking in their neighbourhoods, which underscores the essential role of the built environment in shaping social relations [71]. This finding aligns with Lefebvre’s [8] concept of lived space to suggest that fostering social environments can enhance walking experiences, and consequently public health outcomes.

Understanding how the built environment influences walking behaviour is crucial for developing effective health promotion strategies that encourage neighbourhood walking as a vital component of an active lifestyle [36]. The participants emphasized the importance of road maintenance and community upkeep to reveal that neighbourly behaviours play a pivotal role in fostering a positive walking experience. In the absence of regular maintenance and upkeep, it is inevitable that the quality of pedestrian mobilities of certain groups (e.g., older adults, people with disabilities, and the parents of young children) would be diminished. This finding is especially relevant in light of evidence that neglecting these aspects may deter residents from engaging in urban walking, despite its low-risk profile [2].

Our findings also highlight the critical role community members play in maintaining safe and inviting neighbourhood spaces [55]. A diminished sense of safety can have detrimental effects on specific populations by exacerbating social exclusion and undermining the health benefits associated with walking [2,37]. The participants’ attentiveness to their surroundings enabled them to notice unexpected events and changes in the built environment [28]. While these observations were often secondary to their walking experiences [4], both the top-down and bottom-up interventions were acknowledged as impactful. These initiatives aim to enhance the aesthetic quality of neighbourhood spaces and walking routes through natural and artistic elements. The participants generally agreed on the positive influence of natural elements in public spaces, which aligns with Leavell et al.’s [41] assertion that urban green spaces can strengthen community ties and improve mental health (see also [43,45,46]).

This study identified two types of urban interventions that effectively foster social connections among inhabitants: (1) sanctioned projects that invite community engagement in public spaces (e.g., community events); and (2) community-led changes (e.g., neighbourhood beautification efforts). Both categories may be considered public space animation projects [16] that facilitate sociable encounters and a sense of shared presence [39,72]. The community-driven nature of the latter, by virtue of the differences in the execution stage, holds greater potential for enhancing collective experiences and fostering a sense of belonging.

By examining the modifications in public spaces in tandem with the changes in private properties, we reveal how visibility, content, and location influence the relational dynamics among urban inhabitants. While the appropriation of public space—an integral element of the right to the city [24]—is rarely enacted outside the territorial (and social) boundaries of neighbourhoods, the participants exhibited a heightened sense of safety and agency in familiar environments. This understanding can guide public health strategies aimed at promoting inclusivity and engagement in urban public spaces, as individuals are generally more enthusiastic about enacting changes in areas where they feel secure. However, the tendency of urban inhabitants to confine their interventions in public spaces to their neighbourhoods may also be interpreted as an outcome of the long-lasting tension between city administrations and urban inhabitants in which the former has traditionally aimed to control the latter through policies and development-focused initiatives [8]. Therefore, policies and strategies to extend this sense of security to the broader urban environment (e.g., legacy parks and public squares that are occupied from urban inhabitants living in different neighbourhoods) would help transcend neighbourhood-level peculiarities to potentially become city-level differentiating factors.

This study also highlights that while bottom-up interventions may not always be viewed positively, they remain vital for effective placemaking [27]. The acceptance of these changes within neighbourhoods can dictate their legitimacy and impact [26]. Unlike the more rigid control exerted by political authorities, neighbourhoods can organically establish their own norms and expectations, potentially leading to either restrictive or empowering outcomes [22]. This dynamic illustrates how playful engagement can become an identity marker for neighbourhoods, which may inadvertently result in exclusionary practices for those who do not conform [26]. Recognizing the importance of inhabitant-led changes in the built environment and its connection to social cohesion, and thereby to improved wellbeing urges planners and city administrators to position inhabitants as placemakers exerting their right to the city [24], instead of passive actors in everyday life of the city. This way, it would be possible to minimize the tensions between formal urban interventions (i.e., how spaces are conceived by the authorities) and the urban inhabitants’ everyday lives (i.e., how spaces are lived and experienced by urban inhabitants) [8]. However, while cities often exhibit certain levels of diversity, this diversity does not necessarily entail diverse neighbourhoods. For example, suburban neighbourhoods in KW are mostly composed of detached and semi-detached houses catering to families, overlaying the demographic structure of the neighbourhood with a certain homogeneity, which surely has a reflection on our sample.

Architectural features, such as front porches, emerged as the facilitators of social interactions between the residents and the passersby, while backyards were perceived as socially isolating. This raises important questions regarding the new urbanist claim that improved visual and auditory connections between houses and streets enhance social interactions [73,74]. Given that data collection coincided with the COVID-19 restrictions, the observed dynamics may not fully reflect typical social interactions [6]. Increased time spent at home likely altered the interaction patterns both with the built environment and among the urban inhabitants, prompting further inquiry into whether these changes would persist in normal circumstances.

Interestingly, some participants modified their backyards to increase visibility and accessibility to neighbours, indicating potential for enhanced social connections that could benefit community health [6,40]. Such modifications may contribute to a socially connected neighbourhood, positively impacting residents’ overall health and wellbeing. In light of such observations, urban planning frameworks that offer some flexibilities in how the built environment can be altered would foster inhabitants’ sense of belonging and thereby their overall wellbeing.

Ultimately, this study underscores the complex interplay involving urban design, social interactions, and public health in neighbourhoods. By recognizing the subjective nature of these relationships and emphasizing the importance of safety and visibility, urban planners and public health practitioners can better strategize to promote walking as a collective and inclusive activity that enhances community wellbeing.

## 6. Conclusions

In our research on social interactions of urban inhabitants facilitated by their neighbourhood walks and their interplay with the characteristics of the built environment, four key points emerge. First, neighbourhood walks have an important function in monitoring the conditions and changes in the social and built environments in urban areas. The changes in the built environment, irrespective of their scale, not only have a profound influence over the self-evaluated quality of one’s walks, but they also are a major constituent of continuous placemaking efforts in improving quality of life and wellbeing. Second, the attentiveness of the inhabitants to the publicly visible areas of their private spaces holds a significant potential for facilitating social interactions. This possibility underscores the role of these areas as extended social infrastructure that physically links the private realm of homes with the parochial realm of the neighbourhood and are conducive to social cohesion in neighbourhoods and the perceived social connectedness of inhabitants. Third, although urban inhabitants play with the city by appropriating public spaces in their neighbourhoods [26], these appropriations do not typically transcend neighbourhoods’ territorial boundaries—the right to the city is thereby operationalized as the right to the neighbourhood. A sense of safety and familiarity is decisive in inhabitants’ interventions in the public/parochial realm of their neighbourhood. Fourth, except for rare instances, such appropriations do not typically lead to collective participation, which is restricted primarily to programmed activities undertaken by organizations, which means that urban playfulness is predominantly conceived at the administrative level, thereby consolidating the power of formal administrations over the relationalities that emerge in the public realm of cities with relatively limited resistance from urban inhabitants. While our findings indicate the various ways with which inhabitants exert their right to the city, city administrations also have an important role in facilitating urban interventions to support social connectedness whose positive impacts on communities’ health and wellbeing lead to improved social cohesion.

These findings, however, are subject to some limitations. First, recognizing the importance of different cultural peculiarities on perceptions of neighbourhoods and neighbours, future research in cities with different historical, cultural, and political experiences than our mid-sized North American example would complement this research and inform scholars and practitioners about the different understandings of the right to the city and its spatial ramifications. Each context is unique in its socio-spatial dynamics—urban interventions’ long-term outcomes, particularly as they relate to health and wellbeing, are inseparable from their context.

Second, this research elicited qualitative data from a relatively homogenous demographic group. Gaining a deeper understanding of how neighbourhood walking facilitates interactions for diverse groups (e.g., in terms of ethnicity, race, household income, and age) would both allow for addressing the issues specific to these communities and help identifying the differences in how urban inhabitants connect with their neighbourhoods (and perhaps more crucially, do not). The social, cultural, and economic differences among these groups inadvertently mean that how they interact with a city’s built environment and their fellow inhabitants (or visitors) would vary significantly. Therefore, identifying these differentials would also guide policymakers, city administrators, and urban planners in both improving a sense of belonging and social cohesion and how inclusive public spaces can be built and maintained, both through permanent design features and the temporary animation of public spaces on neighbourhood and city levels.

Third, as mentioned previously, COVID-19-related restrictions surely had an impact on the relationships the urban inhabitants cultivated in their neighbourhoods, as individuals spent more time in close proximity to their residences and walking was one of a few options to stay social [6]. Investigations of how neighbourhood walks influence social interactions and the perception of the built environment in the absence of such restrictions is, therefore, recommended for future research. Moreover, the cross-sectional nature of this research would be complemented by longitudinal investigations of neighbourhood (and more broadly, urban) walking behaviour and the social interactions therein to help researchers uncover the changing trends in how inhabitants navigate their cities.

## Figures and Tables

**Table 1:** Demographic information of study participants.

Demographic Attribute		n	%	Demographic Attribute		n	%
Gender	Female	38	84.4	Residence status	Canadian Citizen	44	97.8
	Male	7	15.6		Permanent resident	1	2.2
Age	70 and above	8	17.8	Living status	Live with parents	2	4.4
	60–69	10	22.2		Own	37	82.2
	50–59	8	17.8		Rent	6	13.3
	40–49	4	8.9	Household size	One-person	5	11.1
	30–39	7	15.6		Two-persons	22	48.9
	29 or less	6	13.3		Three-persons	11	24.4
	Not specified	2	4.4		Four-persons	7	15.6
Ethnicity	Euro-Canadian	43	95.6	Household	10K–24.9K	1	2.2
	Other (Asian)	2	4.4	income in CAD	25K–49.9K	5	11.1
Marital status	Single	5	11.1		50K–74.9K	10	22.2
	In a relationship	5	11.1		75K–99.9K	7	15.6
	Common Law	5	11.1		100K–124.9K	6	13.3
	Married	26	57.8		125K–149.9K	5	11.1
	Divorced	4	8.9		150K<	6	13.3
Education	High school	3	6.7		Not specified	5	11.1
	College	6	13.3	Length of residence	1 year>	6	13.3
	Bachelor’s	19	42.2		1–5 years	8	17.8
	Master’s or higher	17	37.8		6–10 years	7	15.6
					11–15 years	7	15.6
					15 years<	17	37.7
N = 45							

**Table 2:** Sample diary entry.

	Diary Question	Participant’s Answer
1.	Length of the walk	3.4 km
2.	Duration of the walk	50 min
3.	Weather conditions	Cool and sunny
4.	Temperature	5 Celsius
5.	Describe your motivations/purpose for going on a walk? Did you deviate from your original plan? If so, why?	I had to go to the bank, so I walked there. I stopped at a new grocery store on my way back that I hadn’t planned on stopping at, but didn’t deviate from my route to do so. I wanted to walk even though I could have driven because the weather was nice (comparatively) and because I wanted the exercise.
6.	What did you notice about your walking environment? Describe in detail the route you took, and anything you noticed about the landscape of your neighbourhood (a nice garden, a patio, street art, sidewalk or trail, flat/hill, etc.).	I noticed that some of the sidewalks were still un-shoveled making it challenging to traverse in some areas. I walked there down a relatively quiet street and walked back on a busier street. I wanted to see if there were any new shops that had opened in the downtown area since I’m not often on busy streets like King street. I noticed the construction fences around city hall had some colourful artistic banners pinned up on them. Those fences have been up for a while, so it’s nice to see some art being added to the landscape. I noticed that a few stores have closed and that many cannabis stores have opened up, some in close proximity to each other. I saw that a new fancy grocery store has opened up; it had been coming for a while, so I stopped in to see what it offered. I also noticed a new sushi restaurant had opened up, and I paused to look at the menu in the window.
7.	Who did you notice on your walk? Describe your observations of the people you saw, but did not interact with. (Did you wave/nod at anyone, did anyone avoid your path, was there any conflict, etc.)	I saw many people out while I was on my walk. I noticed some people who weren’t walking or heading for a destination but were just hanging out on the streets. Most people, however, appeared to have a destination in mind as they walked. I saw a woman with a walker who appeared to be struggling a bit with navigating the sidewalks due to the snowy conditions. I saw people having to adjust their path due to puddles and slush. At one of the churches, in the front yard, I saw four men sitting around a picnic table visiting and chatting.
8.	Who did you interact with on your walk? Describe in detail your interactions. (Did you speak to anyone, pet a dog, were there any confrontations, etc.)	I didn’t interact with anyone on my walk.
9.	Did you walk with anyone, or any pets? What is your relationship with the person you walked with, do you routinely walk with this person, and did they have any affect on your interactions while walking? If you were not walking with anyone, what were you doing while walking? (Listening to music/podcasts, etc.)	I walked on my own. I listened to a podcast while I walked, and looked in a few windows as I went.
10	Was there anything else noteworthy that you would like to add?	N/A

**Table 3:** The list of questions for the interviews.

Question 1	Why do you go for walks in your neighbourhood? What are your motivations? What influence, if any, has COVID-19 had on your motivation(s) to walk in your neighbourhood or influenced your experience walking in your neighbourhood? (e.g., impacts of physical distancing).
Question 2	What makes for a good (and a bad) interaction during a walk in your neighbourhood? Can you think of a story of an interaction on one of your walks that stands out? Thinking about your neighbourhood walks, talk to me about your interactions with the various kinds of people. How do they contribute to your neighbourhood walking experience? What else, if anything, do they contribute to?
Question 3	How do you make sense of your walking diary entries? What did this exercise reveal about neighbourhood walking to you? Drawing on your walking diary, can you elaborate on, People you encountered, but did not interact with? The people you encountered and interacted with? The person/people/dog(s) you walked with? What happens when you’re walking with others?
Question 4	How do you make sense of your map? What did the mapping exercise reveal about neighbourhood walking to you? Drawing on your mapping exercise, can you elaborate on the kinds of people you encounter (i.e., friends and family, acquaintances, familiar strangers, nodding relationships)?
Question 5	How important, if at all, is connecting with others during your neighbourhood walks? Are there people you avoid or have negative interactions with during your walks? Please expand.
Question 6	In what ways, if any, has neighbourhood walking strengthened your connection to the people you encounter while walking? Can you tell me a story of a relationship you have with someone that was strengthened because of neighbourhood walking?
Question 7	In what ways, if any, have the relationships you developed while walking in your neighbourhood enabled you to draw on neighbours for help or support?
Question 8	What did this study tell you about your experiences walking in your neighbourhood?
Question 9	Is there anything else you would like to add?

## Data Availability

Restrictions apply to the availability of the data to protect the anonymity and privacy of the participants. Requests to access the data should be directed to Troy D. Glover.
